# Paramagnetic artifact and safety criteria for human brain mapping

**DOI:** 10.1186/1476-5918-4-5

**Published:** 2005-05-07

**Authors:** Akitoshi Seiyama, Junji Seki, Mari Iwamoto, Toshio Yanagida

**Affiliations:** 1 Brain Information Group, Kansai Advanced Research Center, Communications Research Laboratory, 588-2 Iwaoka, Nishi-ku, Kobe, Hyogo 651-2492, Japan; 2 Division of Physiology and Biosignaling, Osaka University Graduate School of Medicine, 2-2 Yamadaoka, Suita, Osaka 565-0871, Japan; 3 Department of Biomedical Engineering, National Cardiovascular Center Research Institute, 5-7-1 Fujishiro-dai, Suita, Osaka 565-8565, Japan; 4 Department of Obstetrics and Gynecology, Ehime University, School of Medicine, Shizukawa, Shigenobu-cho, Onsen-gun, Ehime 791-0295, Japan; 5 Soft Biosystem Group, Graduate School of Frontier Biosciences, Osaka University, 2-2 Yamadaoka, Suita, Osaka 565-0871, Japan

## Abstract

Biological effects of magnetic field and their safety criteria, especially effects of gradient magnetic field on the cerebral and pulmonary circulation during functional brain mapping are still unclear. Here we estimated that magnetically induced artifacts for the blood oxygenation level- and flow- based functional magnetic resonance imaging are less than 0.1%, and disturbance in the pulmonary circulation is less than 1.3% even if the field strength of magnetic resonance system is risen up to 10 tesla. These paramagnetic effects are considered to be small and harmless during human brain mapping.

## Introduction

Functional magnetic resonance imaging (fMRI) has become a vital tool for human brain function studies and medical diagnosis. Magnetic field strengths are expected to rise from the current 1.5 to 7 tesla (T) or further to achieve higher spatial resolution and signal-to-noise ratio, although biological effects of magnetic fields and their safety criteria for human subjects are still unclear. Biological effects of magnetic fields depend mainly on 1) the field strength and its gradient, 2) area and duration time of exposure to the field, and 3) the static or dynamic properties of magnetic field [1]. The threshold of time-varying magnetic fields to human exposure is a frequent theme and examined experimentally and theoretically [1,2], because they might produce cardiac and peripheral nerve stimulation, heating, and magneto-phosphene during exposure. Furthermore, movement of subjects or patients within a static magnetic field produces sensory effects such as vertigo, nausea, and a peculiar metallic taste [3].

On the other hand, the static magnetic field has a potential to affect the blood flow through the following three mechanisms: 1) magnetohydrodynamic action, which is theoretically predicted to decrease the aortic blood flow velocity by 10% under homogeneous magnetic fields at about 5 T [4], 2) diamagnetic anisotropic interaction, which modifies the orientation of sickled and normal erythrocytes at homogeneous magnetic field of 0.35 T[5] and 4 T [6], respectively, and 3) paramagnetic interaction under a strong spatial gradient of magnetic field, which was applied to separate paramagnetic erythrocyte from whole blood [7].

## Discussion

### Paramagnetic interaction between gradient magnetic fields and flowing erythrocytes

**Figure 1:**
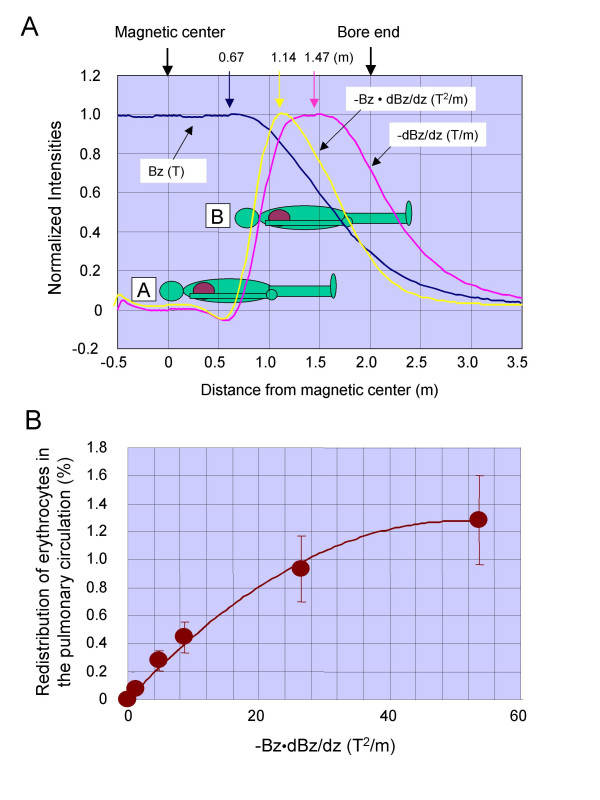
Paramagnetic effects of gradient magnetic fields on the pulmonary circulation during human brain mapping. (A) Magnetic field strength (Bz), and calculated field gradient (-dBz/dz) and force product (-Bz·dBz/dz) along the centerline of the magnet (see '**Supplementary Method 2' in Additional file 1**). Maximal values of -dBz/dz at Bz = 1.5, 3.0, 4.0, 7.0 and 10 T were 1.03, 2.06, 2.75, 4.81 and 6.87 T/m, respectively (at z = 1.47 m). Maximal values of -Bz·dBz/dz at Bz = 1.5, 3.0, 4.0, 7.0 and 10 T were 1.21, 4.84, 8.60, 26.40 and 53.75 T^2^/m, respectively (at z = 1.14 m), which are well covered by the range of our experiment shown in Fig. 1B. The head and lung of a healthy volunteer, whose height is 1.75 m, head length is 0.25 m, length of epigastric fossa from top of head is 0.5 m, were positioned at the magnet's center (A: z = 0 m) and at maximal of -Bz·dBz/dz (B: z = 1.14 m), respectively. (B) Maximally estimated redistribution of erythrocytes estimated in the pulmonary circulation at position B (see 'Supplementary Method 3' in Additional file).

To estimate maximal effects of the gradient magnetic field, which is applied to the human in MR measurements, on the cerebral and pulmonary microcirculation, we examined the effects of gradient magnetic fields on erythrocyte flow in a model of branched blood vessels with Reynold's number as small as that for blood flow in capillaries and venules (see **'Supplementary Method 1' in Additional file **1), which are major contributors to signal changes in fMRI. An inhomogeneous magnetic field (field strength and spatial gradient product: up to 60 T^2^/m) was transversally applied to the blood flow using an electromagnet whose direction of magnetic field is perpendicular to the vessel axis (see Fig. 1 of Ref. [8]), which covered the gradient field of 10-T MRI system (see Fig. 1A). We observed that the spatial distribution of 70% oxygenated erythrocytes flowing in the branched vessel deviated in proportion to the product of magnetic field strength and its gradient up to 20 T^2^/m, above which the deviation saturated. On the other hand, fully oxygenated erythrocytes flow showed no deviation. The deviation of spatial distribution of 70% oxygenated erythrocytes can be attributed to paramagnetic attraction by an inhomogeneous magnetic field [8,9].

### Effect of gradient magnetic fields on the pulmonary circulation

First, we estimated the maximal effects of the deviation of erythrocyte distribution on the pulmonary blood flow, which could cause a ventilation perfusion mismatch (Fig. 1B). When a volunteer was positioned under the maximal inhomogeneous magnetic field (position B in Fig. 1A), the deviation of erythrocyte distribution in the pulmonary blood flow increased with increases in the product of field strength and its gradient but it saturated above |Bz·dBz/dz| > 40 T^2^/m (Fig. 1B). However, the estimated value of deviation (maximally ca. 1.3%) was small and its effect on the ventilation perfusion mismatch is negligible even under the maximal inhomogeneous magnetic field generated by the 10-T system.

### Effect of gradient magnetic fields on the cerebral microcirculation

**Figure 2:**
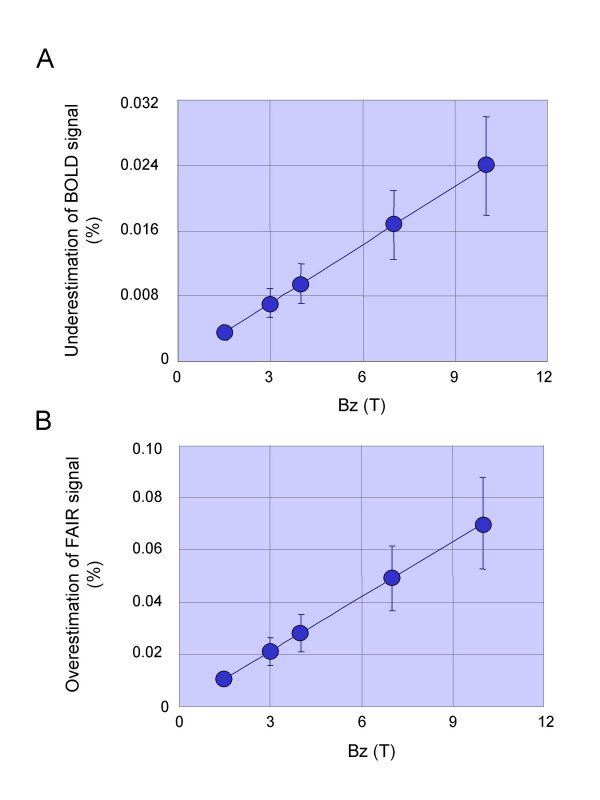
Effects of magnetic field under gradient echo echo planar imaging (GE-EPI) on the cerebral microcirculation. (A) Maximal effects of magnetic field under GE-EPI on BOLD signal at position A during functional brain mapping at slice gradient = 40 mT/m for all MR systems (see **'Supplementary Method 4' in Additional file 1**). (B) Maximal effects of magnetic field under GE-EPI on FAIR signal at position A during functional brain mapping at slice gradient = 40 mT/m for all MR systems (see **'Supplementary Method 5' in Additional file**).

Next, we estimated maximal effects of such deviation of erythrocyte distribution on signal changes in functional neuroimaging based on the commonly used blood oxygenation-level dependent (BOLD) (Fig. 2A) and flow sensitive alternating inversion recovery (FAIR) (Fig. 2B) techniques. The gradient magnetic fields produced underestimation of BOLD and overestimation of FAIR signals almost linearly with the increases in the magnetic field strength. However, the values (0.024% for BOLD and 0.07% for FAIR) were quite small and these artifacts are negligible even for the 10-T system.

## Conclusion

Our results suggest that paramagnetic artifacts in the functional neuroimaging and disturbance in the pulmonary microcirculation during magnetic resonance imaging are quite small using the MR system with field strength up to 10 T.

## Authors' contributions

AS conceived, designed and coordinated the study, carried out the experiments, and drafted the manuscript. JS participated in the design and coordination of the study. MI participated in the experiments. TY participated in the design and coordination of the study, and directed it. All authors read and approved the final manuscript.

## Supplementary Material

Additional File 1 Supplementary Method 1: Experimental Setup. Supplementary Method 2: Estimation of magnetic fields. Supplementary Method 3: Estimation of redistribution of erythrocytes in the pulmonary circulation. Supplementary Method 4: Estimation of paramagnetic artifacts in BOLD signal. Supplementary Method 5: Estimation of paramagnetic artifacts in FAIR signal Click here for file
