# Supplementary material for: Paramagnetic artifact and safety criteria for human brain mapping
Source: Dyn Med. 2005 May 7;4:5. doi: 10.1186/1476-5918-4-5 (PMC1142518; doi:10.1186/1476-5918-4-5)
Supplement: Additional File 1 — Supplementary Method 1: Experimental Setup. Supplementary Method 2: Estimation of magnetic fields. Supplementary Method 3: Estimation of redistribution of erythrocytes in the pulmonary circulation. Supplementary Method 4: Estimation of paramagnetic artifacts in BOLD signal. Supplementary Method 5: Estimation of paramagnetic artifacts in FAIR signal [file 1476-5918-4-5-S1.doc]

# Additional file

**Supplementary Method 1: Experimetal Setup.** Details in a model of branched blood vessels and generation of gradient magnetic fields are described in elsewhere [1]. In brief, a straight glass vessel with a rectangular side branch was positioned in an inhomogeneous magnetic field generated by an electromagnet. Suspension of human erythrocytes, which are prepared similar to venous blood as 45% of hematocrit and 70% of oxygenation, was allowed to flow through the model vessel with Reynold’s number as small as that for blood flow in capillaries and venules. Displacement or deviation of erythrocyte distribution flowing into the side branch was spectrophotometrically analyzed. The relationship between gradient magnetic field and deviation of erythrocyte distribution was extrapolated to the case of human brain mapping using MRI as follows.

**Supplementary Method 2: Estimation of magnetic fields (Fig. 1A).** Magnetic field strength (Bz), field gradient (-dBz/dz) and force product (-Bz·dBz/dz) along the center line of the magnet were calculated according to the instrumentation of those reported by Schenck et al. [2]. For the calculation, we assumed that the current intensities for 1.5, 3.0, 4.0, 7.0 and 10 T MR system were 85.7, 171.5, 228.6, 400.5 and 571.5 A, respectively.

**Supplementary Method 3: Estimation of redistribution of erythrocytes in the pulmonary circulation (Fig. 1B).** According to the method of Seiyama et al [1], deviation of erythrocyte distribution in the blood (ΔV/V_rest_) was estimated in the pulmonary circulation of the subject positioned at B, assuming the maximal transit time of the blood about 6 s [3].

**Supplementary Method 4: Estimation of paramagnetic artifacts in BOLD signal (Fig. 2A).** According to the method of Seiyama et al. [1], deviation of the erythrocyte distribution in the blood (ΔV/V_rest_) during functional neuroimaging at position A was estimated assuming that the slice gradient is 40 mT/m in spite of the field strength of MR systems and the maximal transit time of the blood in the cerebral circulation is 6 s [4]. Using these parameters, the value (ΔV/V_rest_) was converted to the BOLD signal change, (ΔS/S_rest_)_BOLD_, according to the method of Seiyama et al. [5], (ΔS/S_rest_)_BOLD_ = 0.895·ΔV/V_rest_.

**Supplementary Method 5: Estimation of paramagnetic artifacts in FAIR signal (Fig. 2B).** The above estimated value (ΔV/V_rest_) was converted to the change in FAIR signal by applying Grubb’s equation [6], ΔCBF/CBF_rest_ = 2.63・(ΔCBV/CBV_rest_), and Kim’s [7] equation, (ΔS/S_rest_)_FAIR_ = ΔCBF/CBF_rest_ = 2.63·ΔV/V_rest_.

References

1. Seiyama A, Maeda N, Shiga T: **Analysis of distribution of flowing erythrocytes**

**in a model vessel under an inhomogeneous magnetic field.** *Eur Biophys J* 1996*,*

**25**: 1-7.

1. Schenck JF, Dumoulin CL, Redington RW, Kressel HY, Elliott RT, McDougall IL:

**Human exposure to 4.0-Tesla magnetic fields in a whole-body scanner.** *Med*

*Phys* 1992*,* **19**: 1089-1098.

1. Zavorsky GS, Walley KR, Russell JA: **Red cell pulmonary transit times through**

**the healthy human lung.** *Exp Physiol* 2003, **88**: 191-200.

1. Keller E, Nadler A, Alkadhi H, Kollias SS, Yonekawa Y, Niederer P: **Noninvasive**

**measurement of regional cerebral blood flow and regional cerebral blood**

**volume by near-infrared spectroscopy and indocyanine green dye dilution.**

*NeuroImage* 2003, **20**: 828-839.

1. Seiyama A, Seki J, Tanabe HC, Sase I, Takatsuki A, Miyauchi S, Eda H, Hayashi S,

Imaruoka T, Iwakura T, Yanagida T: **Circulatory basis of fMRI signals:**

**relationship between changes in the hemodynamic parameters and BOLD signal**

**intensity.** *NeuroImage* 2004, **21**: 1204-1214.

1. Grubb RL, Raichle ME, Eichling JO, Ter-Pogossian MM: **The effects of changes in PaCO2 on cerebral blood volume, blood flow, and vascular mean transit time.** *Stroke* 1974, **5**: 630-639.
2. Kim SG: **Quantification of relative cerebral blood flow change by flow-sensitive alternating inversion recovery (FAIR) technique: application to functional mapping.** *Magn Reson Med* 1995, **34**: 293-301.
